# The roles of Eu during the growth of eutectic Si in Al-Si alloys

**DOI:** 10.1038/srep13802

**Published:** 2015-09-02

**Authors:** Jiehua Li, Fredrik Hage, Manfred Wiessner, Lorenz Romaner, Daniel Scheiber, Bernhard Sartory, Quentin Ramasse, Peter Schumacher

**Affiliations:** 1 Institute of Casting Research, Montanuniversität Leoben, Leoben, A-8700, Austria; 2 SuperSTEM Laboratory, SciTech Daresbury Campus, Keckwick Lane, Daresbury, WA4 4AD, UK; 3 Materials Center Leoben Forschung GmbH, Leoben, A-8700, Austria; 4 Austrian Foundry Research Institute, Leoben, A-8700, Austria

## Abstract

Controlling the growth of eutectic Si and thereby modifying the eutectic Si from flake-like to fibrous is a key factor in improving the properties of Al-Si alloys. To date, it is generally accepted that the impurity-induced twinning (IIT) mechanism and the twin plane re-entrant edge (TPRE) mechanism as well as poisoning of the TPRE mechanism are valid under certain conditions. However, IIT, TPRE or poisoning of the TPRE mechanism cannot be used to interpret all observations. Here, we report an atomic-scale experimental and theoretical investigation on the roles of Eu during the growth of eutectic Si in Al-Si alloys. Both experimental and theoretical investigations reveal three different roles: (i) the adsorption at the intersection of Si facets, inducing IIT mechanism, (ii) the adsorption at the twin plane re-entrant edge, inducing TPRE mechanism or poisoning of the TPRE mechanism, and (iii) the segregation ahead of the growing Si twins, inducing a solute entrainment within eutectic Si. This investigation not only demonstrates a direct experimental support to the well-accepted poisoning of the TPRE and IIT mechanisms, but also provides a full picture about the roles of Eu atoms during the growth of eutectic Si, including the solute entrainment within eutectic Si.

The Na modification of eutectic Si was patented 94 years ago (in 1921) by A. Paczki^1^. 44 years later (in 1965)^2^, the detailed mechanism for the modification was still a matter of controversy due to a lack of a detailed microstructure investigation on an atomistic level, although the modification of eutectic Si had by then been widely used in the foundry industry. To date, it is generally accepted that the impurity-induced twinning (IIT) mechanism^3^ and the twin plane re-entrant edge (TPRE) mechanism^4^,^5^ as well as poisoning of the TPRE mechanism^6^ are valid under certain conditions and contribute to the modification process. The IIT mechanism postulates that modifying elements (such as Na, Sr) are adsorbed at ledges on the growing {111}_Si_ surfaces producing frequent multiple Si twinning. The TPRE mechanism proposes that the growth of eutectic Si occurs more readily at the re-entrant edge. Poisoning of the TPRE mechanism assumes that the modifying elements retard the growth of eutectic Si by selectively adsorbing at TPRE and thereby deactivate the growth advantage of the TPRE mechanism. However, there is a lack of direct experimental support for these growth mechanism models. For example, no single Na atomic column has been observed yet in Na-modified alloys, although Na-rich clusters were observed using atom probe tomography (APT) to be located within eutectic Si and at the interface between Al and eutectic Si^7^. Similarly, Sr-rich clusters, rather than single Sr atom columns, were observed using high resolution high angle annular dark field (HAADF) scanning transmission electron microscopy (STEM) imaging and APT^8^,^9^,^10^,^11^. Such kinds of high resolution experimental observation can be certainly used to support the validity of the IIT, TPRE or poisoning of TPRE mechanisms, but a direct experimental observation of single modifying-element-rich atomic columns is still required to truly elucidate their roles in the growth of eutectic Si. Furthermore, the IIT, TPRE or poisoning of TPRE mechanisms cannot be used to interpret all observations accompanying the modification^12^,^13^,^14^,^15^,^16^,^17^, indicating that other factors may also play an important role during the growth of eutectic Si^10^,^11^. For example, Yb^12^ or Ca^13^,^14^ only refine, rather than modify, the eutectic Si, even though Yb or Ca possess a favourable atomic radius ratio (r/r_Si_ = 1.646) according to the IIT mechanism. Furthermore, among the rare earth elements, only Eu can modify eutectic Si to a fibrous morphology^15^,^16^,^17^ and Eu is more likely to combine with Si atoms, forming Eu-rich clustering^17^. For this latter case in particular, there is still a lack of a detailed investigation on the distribution of Eu atoms within eutectic Si (i.e. at the twin plane re-entrant edge or at the intersection of Si facets and respectively twins). Such type of atomistic information is of great importance to elucidate the well-known poisoning of the TPRE and IIT mechanisms.

In this paper, HAADF STEM imaging and electron energy loss spectroscopy (EELS) were used to elucidate the distribution of Eu atoms within eutectic Si. The atomic number of Eu (63) is much higher than that of Al (13) and Si (14), therefore giving a strong contrast in HAADF STEM imaging and providing a favourable case for the observation of single Eu atomic columns, especially when combined with EELS mapping to confirm the chemical identification. First-principle calculations were also performed to elucidate the segregation behaviour of Eu atoms within Si twins and its effect on the Si twinning. The aim of this investigation is to provide direct experimental supports to the well-accepted poisoning of the TPRE and IIT mechanisms, and also to elucidate other roles of Eu during the growth of eutectic Si, including the solute entrainment within eutectic Si.

## Results

### HAADF STEM imaging and EELS

**Figure 1:**
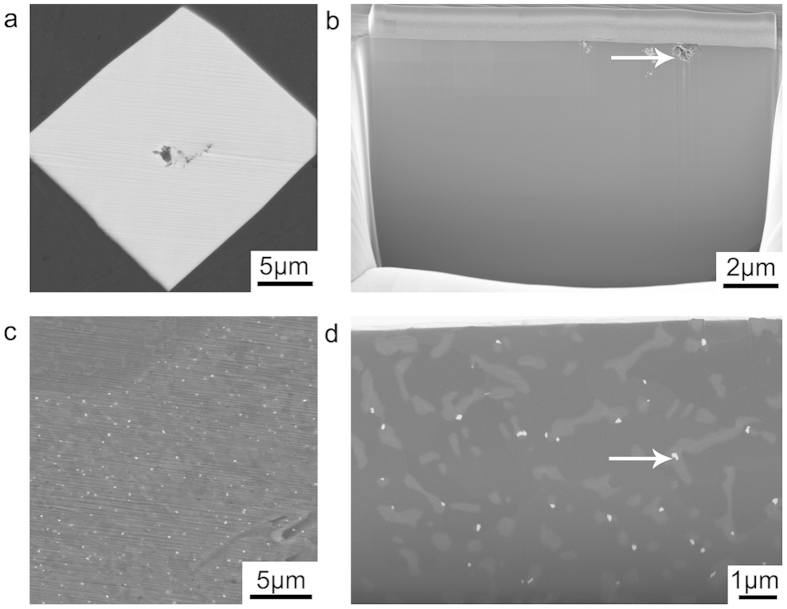
SEM images of the Eu-containing phase in Al-5Si-0.05Eu alloy. (**a**) shows a coarse Al_2_Si_2_Eu phase. (**b**) shows the presence of impurities within the coarse Al_2_Si_2_Eu phase, as marked with a white arrow. (**c**) shows small Al_2_Si_2_Eu phases in the vicinity of eutectic Si. (**d**) shows small Al_2_Si_2_Eu phases within eutectic Si, as marked with a white arrow. The images in (**a**,**c**) were acquired before FIB cutting. While, (**b**,**d**) were acquired after FIB cutting.

Figure 1 shows SEM images of the Eu-containing phases in Al-5Si-0.05Eu alloy. Three types of Al_2_Si_2_Eu phases were observed^17^. Firstly, a coarse Al_2_Si_2_Eu phase was observed, as shown in Fig. 1a. Such type of Al_2_Si_2_Eu phase can be regarded as a pre-eutectic phase formed before the eutectic reaction and resulting from a high Eu concentration (0.05 wt.%). Within the coarse Al_2_Si_2_Eu phase, impurities (e.g. Fe or O) were observed, as marked with a white arrow in Fig. 1b. Secondly, a small Al_2_Si_2_Eu phase was observed in the vicinity of eutectic Si, as shown in Fig. 1c. This type of small Al_2_Si_2_Eu phase can be attributed to the growth of Eu-containing clusters formed ahead of the solidification front during the segregation of the eutectic reaction. Thirdly, a small Al_2_Si_2_Eu phase was also observed within the eutectic Si, as marked with a white arrow in Fig. 1d. Such type of Al_2_Si_2_Eu phase can be attributed to the entrainment and growth of Eu-containing clusters formed within the eutectic Si during the growth of eutectic Si. A detailed investigation of Eu-containing clusters within the eutectic Si is of great importance for revealing the modification mechanism caused by Eu, and is, therefore, a focus of this paper.

**Figure 2:**
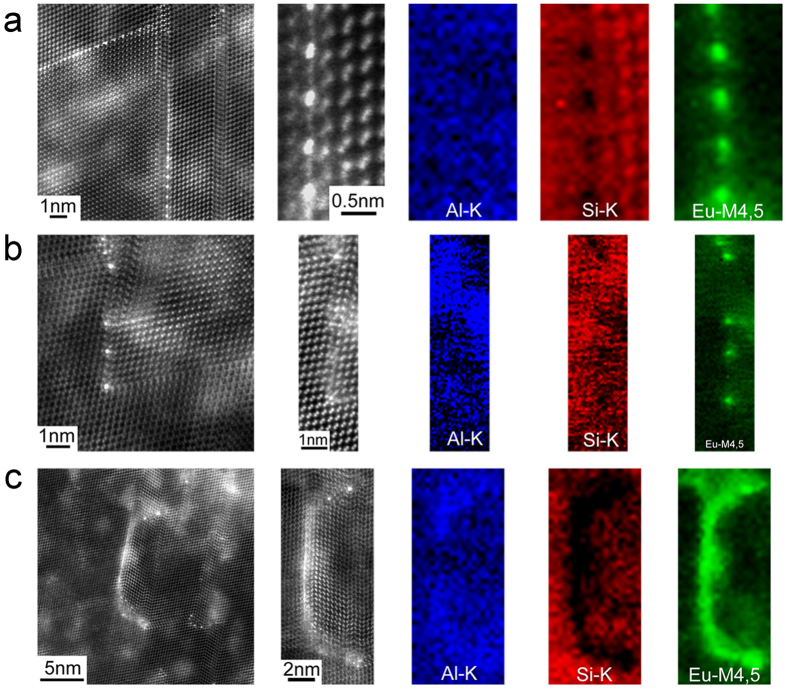
High resolution HAADF STEM images and EELS maps of Al, Si and Eu in Al-5Si-0.05Eu alloy. (**a**) shows Eu atoms between every two Si atomic columns are located at the twin plane re-entrant edge, indicating that poisoning of the TPRE mechanism is active. (**b**) shows Eu-rich atomic columns are located at the intersection of Si facets and respectively twins, indicating that the IIT mechanism is active. (**c**) shows Eu atoms are located ahead of the growing Si twins, forming a continuous Eu-rich layer, indicating that a solute entrainment occurs within eutectic Si.

Figure 2 shows HAADF STEM images and EELS maps of Al, Si and Eu acquired from specific regions within eutectic Si in the Al-5Si-0.05Eu alloy which were pre-identified as Eu-rich. Contrast in HAADF STEM images scales to a good approximation with the atomic number Z of the observed material as ~Z^1.7^ so that columns containing Eu atoms within a Si matrix are clearly distinguishable with a much brighter intensity, an observation also clearly confirmed by the chemical maps obtained with EELS (see methods section).

Apart from Si and Eu, a faint Al signal was also measured within the eutectic Si, as seen in the maps of Fig. 2, provided for completeness. However, the intensity of the Al signal is much lower than that of Si or Eu: the color scale maps are normalised to 1 for convenience, (see methods section), but as can be clearly observed on Figure S1 in the supplementary information provided, which shows a typical spectrum from one of the chemical maps, the relative Al signal is relatively low. The maximum solid solubility of Si in Al is 1.65 wt.% at the eutectic temperature of 577 °C, while the solid solubility of Al in Si is less than 0.0001 wt.% Al at the eutectic temperature^11^. Therefore, the Al concentration within the eutectic Si is expected to be at trace level only. A more complex scenario could occur during solidification, especially at the stage of the growth of eutectic Si, Al may become entrained by overlapping of different eutectic Si, in particular in the presence of modifying agents (e.g. Sr, Na and Eu). The detection of Al is nevertheless more likely due to the fact that the signal is averaged through the thickness of the sample, and some Al matrix surrounding the eutectic Si may remain further in the depth of the lamella; alternatively, although less likely to be the main contributing factor, some Al may have been re-deposited on the surface of the lamella during sample preparation and thinning, giving rise to the observed EELS intensity. Regardless, the Al detected in the chemical maps is unlikely to have any structural effect and the role of Al on the structure and segregation behaviour of the twin boundary is therefore not taken into consideration here.

An extremely interesting case of Eu adsorption was observed in Fig. 2a which shows a twin (more accurately described as a complex twin defect) in which Eu atoms are located approximately between every two Si atomic columns at the twin plane re-entrant edge. This may provide a hint that Eu bonds preferentially to Si atoms rather than Eu atoms, in turn indicating that poisoning of the TPRE growth mechanism is active. Interestingly, some small mis-tilt and lattice deformation were observed on either side of the Eu-rich plane, resulting in non-perfect imaging conditions: this is the case in particular for the image shown in the second panel of Fig. 2a (showing the HAADF contrast acquired simultaneously with the EELS maps), where the Si ‘dumbbell’ structure is less clear on the left-hand side of the defect. This might be understood in terms of local distortions due to steric constraints imposed by the relative size difference of Eu and Si atoms and will be discussed later. As originally proposed in the TPRE mechanism and poisoning of the TPRE mechanism, Si growth occurs more readily at the re-entrant edge and the modifying element retards Si growth by selectively adsorbing at the TPRE and thereby deactivates the growth advantage of the TPRE mechanism. The images presented here provide for the first time experimental evidence for Eu adsorption at the TPRE in this system. Previous reports in related systems showed that (i) rare-earth atoms (La, Sm, Er, Yb, and Lu) can bond to two different attachment locations in Si_3_N_4_^18^ grain boundaries depending on their atomic size, electronic configuration, and the presence of oxygen, and (ii) Y and Yb are enriched at the interface (atomically flatter, only one possible attachment location) between SiC grain and grain-boundary phase in rare-earth (Y and Yb) doped SiC^19^. However, the reason why the Eu atoms seem here to be preferentially located between every two Si atomic columns in Si crystal still remains to be explored, and will be the object of a discussion in a later part of this paper focused on density functional simulations.

Figure 2b shows that a number of Eu atoms are located at the intersection of Si facets and respectively twins, providing direct experimental evidence that the IIT mechanism is also active. As originally proposed in the IIT mechanism, the modifying element is adsorbed on the growing {111}_Si_ surfaces producing frequent Si twinning. Indeed, single Eu atomic columns, rather than Eu-containing clusters, were observed, for the first time, to be adsorbed on the growing {111}_Si_ surfaces. Furthermore, every {111}_Si_ is decorated by a single Eu-rich atomic column, and the growth direction of eutectic Si is at this point also changed from one {111}_Si_ to another {111}_Si_, indicating that the presence of Eu atoms on the growing {111}_Si_ surfaces can promote the formation of multiple Si twinning. However, it is still of great necessity to elucidate the interaction between Eu atoms and Si atoms, which will be fully interpreted in the density functional simulation section below.

Eutectic Si grows along {111}_Si_ planes via poisoning of the TPRE mechanism (Fig. 2a) and IIT mechanism (Fig. 2b). During the growth of eutectic Si, Eu and Al solute will segregate ahead of the solidification interface (k_Al_ < 1 and k_Eu_ < 1). A solute redistribution of Eu and Al, and thus an enrichment of Eu and Al ahead of the solidification front may occur. During further growth of eutectic Si, the adsorption of Eu atoms at the intersection of Si twins (Fig. 2b) and/or along the <112>_Si_ growth direction (Fig. 2a) can yield further re-entrant edges of Si twinning or a change of stacking sequence of Si twinning. The growth of eutectic Si will re-start from another {111}_Si_ plane. Once the {111}_Si_ planes fold on each other, the solute impingement and subsequent entrainment of the segregation fields result in local compositions favourable for subsequent formation of intermetallic phase.

Figure 2c illustrates this behaviour and shows that Eu atoms can also be located ahead of the growing Si twins, indicating that a solute entrainment occurs within eutectic Si. The continuous Eu-rich layer is only one atomic plane in thickness. No clear atomic structure is discernible along this Eu-rich plane, which is different from the discrete Eu-rich atomic columns (where each Eu-rich atomic column was surrounded by Si atomic columns rather than adjacent to further Eu-rich columns), as shown in Fig. 2a. Furthermore, no significant Si twinning was observed along the continuous Eu-rich layer, strongly indicating that the formation of the continuous Eu-rich layer is caused by the solute entrainment during the growth of eutectic Si, rather than the poisoning of the TPRE growth mechanism. Therefore, such a continuous Eu-rich layer caused by the solute entrainment is not believed to promote the formation of the parallel Si twins (Fig. 2a) or the multiple Si twins (Fig. 2b). It can be regarded as an “artefact” during the growth of eutectic Si, instead of an active factor affecting the modification. During the subsequent cooling, the solute entrainment forms an Al_2_Si_2_Eu phase if the cooling rate is low enough or if the segregation time and length is sufficient enough, as shown in Fig. 1d.

Similar to the formation of Al_2_Si_2_Eu phases, the formation of Al_2_Si_2_Sr and AlSiNa phases have been also observed within eutectic Si and in the vicinity of eutectic Si^7^,^8^. However, these phases are, in fact, artefacts formed during the growth of eutectic Si, rather than the real modification mechanism^11^, because of the following facts: (i) a few different elements such as Ca^13^,^14^ and Yb^12^ were also usually observed in the Al_2_Si_2_Ca and Al_2_Si_2_Yb phases. These elements only refine the eutectic Si, strongly indicating that the formation of AlSiX type phases alone cannot be responsible for modification of eutectic Si. (ii) in the present investigation, only single Eu-rich atomic columns or Eu-rich continuous layers with one atomic plane in thickness were observed within the eutectic Si, strongly indicating that the adsorption of the single atomic columns of modifying agents (e.g. Eu atoms) ahead of solidification front, rather than the formation of AlSiX type phases, is one of the dominant factors affecting the modification of eutectic Si.

### Calculations based on Density Functional Theory

Based on the experimental results (Fig. 2), the adsorption or segregation of Eu atoms can be clearly elucidated. However, it is still very challenging from the experimental results only to obtain information about the interaction between Eu atoms and Si atoms, the structure of Si twin boundaries as well as the segregation energy to these Si twin boundaries (TB) with different Eu coverage. To address these questions, calculations based on density functional theory (DFT) were carried out.

As can be clearly seen from the experimental images, a number of complex line defects within the eutectic Si are decorated by Eu-rich atomic columns, including twins and ‘twin-like’ defects. As mentioned above, even in the more simple cases, some small imperfections in the imaging conditions on either side of the twin can result in a level of structural complexity and local lattice distortion. Because of the complexity of these structures, and because a full model of the defects could only be obtained through three-dimensional characterisation which would clearly be difficult here, the DFT study focused on the simplest case where Eu would segregate at a pure TB, to inform and rationalize the experimental observations.

The Si TB structure was modelled as a Σ3<110> {111} TB. With this structure as a starting point, Eu atoms are placed at different positions in the TB to investigate the energetics of periodic and planar arrangements of Si atoms in the TB in response to the presence of Eu. The position of Eu along the TB is approximately based on the experimental observation using HAADF STEM image, whereby Eu-rich columns are never adjacent to each other. Such type of Eu segregation is also consistent with the proposed simultaneous occurrence of the poisoning of the TPRE and IIT growth mechanisms. Therefore, the starting models for the DFT calculations are constructed assuming that Eu atomic columns are segregated along the TB. It should be noted that for simplicity the models assume pure Eu columns, even though the EELS data cannot exclude the fact that these columns are simply highly enriched in Eu (thus still containing an insignificant concentration of Si).

**Figure 3:**
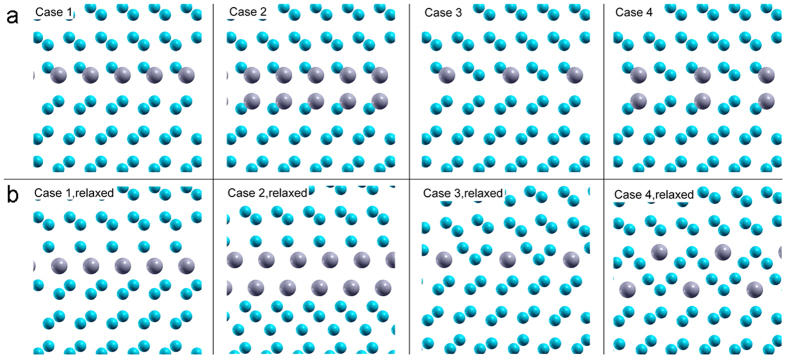
(**a**) Starting structures and (**b**) relaxed structures for four different cases of Eu atoms within eutectic Si. In Case 1, all Si atoms on one side of the TB have been substituted with Eu atoms which corresponds to a full coverage of Eu atoms on one side of the Si twin. In Case 2, all Si atoms on both sides of the TB have been substituted with Eu atoms which corresponds to a full coverage of Eu atoms on both sides of the Si twin. Similarly, Case 3 corresponds to half coverage of Eu atoms on one side of the Si twin, and Case 4 corresponds to half coverage of Eu atoms on both sides of the Si twin.

Four different cases are shown in Fig. 3a, where Eu atoms are shown in grey and Si in blue. In Case 1, all Si atoms on one side of the TB have been substituted with Eu atoms which corresponds to a full coverage of Eu atoms on one side of the Si twin. In Case 2, all Si atoms on both sides of the TB have been substituted with Eu atoms which corresponds to a full coverage of Eu atoms on both sides of the Si twin. Similarly, Case 3 corresponds to half coverage on one side of the Si twin, and Case 4 corresponds to half coverage on both sides of the Si twin. Due to periodic boundary conditions, Eu atoms are aligned in a column in the direction into the picture plane. For every case, the geometric structure is obtained based on a γ-surface approach. The final structures corresponding to the lowest TB energy are shown in Fig. 3b.

Eu was found to induce significant structural relaxations in the TB. In Case 1 and Case 2, Eu atoms are shifted into the center of the Si twin, pushing Si atoms out of the TB. The bottom section of the structure in Case 1 is translated rightwards to adjust the bond angles of the next-neighbouring Si atoms. For Case 2, an even more significant structural rearrangement was observed in the lower part of the twin. For Case 3 and Case 4, both Eu atoms and Si atoms occupy the TB due to the half coverage. The geometric arrangements of these two cases do not resemble the original un-relaxed twin structure any more, but reveal new patterns, especially for Case 4, which can be regarded as a localised two-dimensional Eu-Si phase.

In order to address which geometric variant is energetically preferred and compare calculated structures with experimentally observed structures, the segregation energy of one Eu atom to the TB was calculated for all four cases using Eq. (1)^20^:


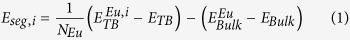


where 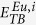 is the total energy of case i containing *N*_*Eu*_ Eu atoms at the unit cell, *E*_*TB*_ is the total segregation energy of the pure Si TB, *E*_*Bulk*_ is the total energy of a large Si bulk cell, and 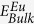 is the energy of this bulk cell where one Si atom is replaced by one Eu atom and a structural relaxation was carried out. The segregation energy gives the energetic gain that a solute experiences when moving from the bulk into the TB. Note that according to Eq. (1) the segregation energy is negative if an element prefers to segregate to the TB. When the number of available Eu atoms is not enough to fully occupy all TBs, the total energy of the system is minimal if every Eu atom minimizes its segregation energy. The experimental observation in Fig. 2 reveals that some TBs are still unoccupied, supporting, hence, the validity of our energetic argument based on the segregation energy. The resulting segregation energies are listed in Table 1. For all cases negative segregation energies are obtained, indicating that segregation of Eu atoms to the TB is energetically preferred. Segregation is strongest for Case 3 and Case 4, which exhibit segregation energies of about −4 eVs. Case 1 reveals intermediate segregation energy of −3 eVs, while Case 2 reveals the weakest segregation energy (about −2 eVs). The bond lengths for Eu-Si extracted from the corresponding relaxed structures are also listed in Table 1. The bond lengths are in the range from 2.92 to 3.09 Å, which is in good agreement with reported experimental measurements (Eu-Si: 3.081–3.12 Å)^17^. Interestingly, there is no correlation between bond length and segregation energy, indicating that bond angles may dominate the energetics, which is consistent with the picture of a covalent bond expected for Si. In this connection, the structural pattern of Eu and Si atoms found in Case 3 and Case 4 was highlighted, which appears to cause to large energetic stabilization. The fact that these structures with mixed Eu-Si occupation at the TB (i.e. Case 3 and Case 4) are energetically preferred is fully consistent with the experimental observation in Fig. 2a where Eu atoms are located between every two Si atomic columns at the twin plane re-entrant edge, and the previous report^21^ that Eu atoms are adsorbed at the bridge/cave sites (half the Eu atoms) and the pedestal/valley bridges (the other half the Eu atoms) on the Si (100) − 2 × 3 surface. It has also been reported in^21^ that the atomic structure model of Si (100) − 2 × 3 − Eu is different from that of Si (100) − 2 × 3 − Na^22^ and Si (100) − 2 × 3 − Sr^23^, although these three elements can modify eutectic Si, indicating that different adsorbate atoms may have different interaction with Si atoms. It should be noted here that our present investigation focus on the Σ3<110> {111} TB, rather than Si (100) surface. To our knowledge, no previous research on the Σ3<110> {111} TB has been reported.

In order to shed more light onto the bonding mechanism of Eu in Si further DFT investigations were carried out. The detail is provided in the supplementary information. Here, only the most important results are summarized: (i) The elastic strain energy induced by dissolving an isolated Eu atom in Si can be estimated to amount to about 0.4–0.5 eVs per Eu atom on the basis of a linear elastic model. It is a relatively small contribution compared to the segregation energy of −4 eVs, which, hence, is unlikely to be caused by the relief of elastic strain, but rather arises due to strong chemical interactions between Eu and Si. (ii) Eu is divalent, meaning that it can be in a +3 or +2 oxidation state. The results discussed above (Table 1 and Fig. 3) are valid for Eu in the +3 oxidation state. Repeating the same calculations for Eu in the +2 oxidation state does not lead to decisive differences in terms of segregation energy and geometry of the four considered cases. (iii) To investigate the role of the twin geometry for the segregation behavior, the bulk analogon of Cases 1–4 was also calculated. Again, very similar results are obtained for the four cases, indicating that it is the specific planar arrangement that leads to the large energetic rather than the distorted bonding environment at the twin interface. This indicates that the reason why Eu atoms are enriched at the twin is not solid state segregation in the common picture where solute atoms diffuse through the solid and get trapped at the TB due to a more favorable bonding environment. Rather, during solidification, Eu atoms are adsorbed preferentially at the TPRE and, as twin grows, the Eu atoms are incorporated into it column by column. If planar rearrangements could be created in the bulk by some procedure, they would be equally stable as the ones at the TB.

## Discussion

Although a comparison of the experimental HAADF STEM images, and in particular Fig. 2a,b, with DFT-calculated models is not expected to yield a perfect one-to-one correspondence due to the simplified model considered for the simulations, a moderately good level of agreement was observed with Case 3, Fig. 3b. After relaxation this model exhibits a structure whereby Eu-rich columns alternate with Si columns, indicating a preference for Eu to bond with Si rather than cluster and forming what could be described as a local Eu-Si phase. The Eu atoms were also observed after relaxation to have migrated away from their initial twin plane termination position into these more complex structural units, a trend also apparent in the experimental data. This is also the case for Case 4, Fig. 3b, which DFT calculation indicates is also energetically highly stable, although there the correspondence with structures observed experimentally is more tenuous. Nevertheless, these simple TB models and the observation of similar structures in experimental images reveal important aspects of the Eu segregation within eutectic Si. Firstly, the incorporation of Eu atoms at twins is accompanied by a significant local modification of the atomic structure. Again, this is most clearly seen in Fig. 2a, where Eu columns are positioned partially ‘in-between’ surrounding rows of Si-dumbbells while similar local distortion can be seen in the relaxed Case 3 and Case 4 structures. Overall, this is indicative of a significant amount of strain being associated with the incorporation of Eu atoms. Secondly, the fact that Eu atoms were observed both experimentally and in the calculated model to adopt positions that do not correspond to the twin terminating plane might be understood in terms of steric constraints imposed by the relative size difference of Eu and Si atoms.

Despite the lack of full one to one correspondence with the DFT models, the present HAADF STEM and DFT investigation reveals, for the first time, three different roles of Eu atoms during the growth of eutectic Si: (i) the adsorption at the intersection of Si facets, inducing IIT mechanism, (ii) the adsorption at the twin plane re-entrant edge, inducing poisoning of the TPRE mechanism, and (iii) the segregation ahead of the growing eutectic Si, inducing a solute entrainment within eutectic Si. Clearly, Eu has an important effect on the growth of eutectic Si. Therefore, the following discussion mainly focuses on the role of Eu atoms during the growth of eutectic Si in Al-Si alloys, in particular to the adsorption kinetic of Eu atoms on {111}_Si_ growth facets.

Si twins can be formed via three different ways: (1) natural TPRE, (2) the adsorption of Eu atom in the bulk Si, promoting the formation of Si twins, and (3) the adsorption of Eu atom at the intersection of Si twins (Fig. 2b) and/or along the <112>_Si_ growth direction, inducing poisoning of TPRE mechanism and IIT mechanism, and thereby promoting the formation of Si twins with higher number densities. The absence of Eu atoms along certain Si twins can be attributed to the fact that (i) the Si twin grows *via* natural TPRE, and thereby no adsorption of Eu is required, and (ii) desorption of Eu may occur during the growth of eutectic Si. It is well-accepted that adsorption and desorption are essential elementary processes because adsorption activates the decisive chemical bonds of the reactants and desorption removes the reaction products from the surface. In a typical adsorption process, the adsorptive and the adsorbent often undergo a complex chemical reaction. The chemical and physical property of the adsorbate is not always just the sum of the individual properties of the adsorptive and the adsorbent, but often represents a phase with novel properties.

For a successful adsorption of Eu atoms on {111}_Si_ growth facets, the Eu atoms have to remain adsorbed for a sufficiently long period of time so that they are not segregated out of the Si as Si has a very constrained solubility for Eu. This can be used to interpret the fact that not all Si twins are decorated by Eu atoms because of the absence of Eu atoms on {111}_Si_ growth facets (Fig. 2a). Once Eu atoms have been adsorbed on {111}_Si_ growth facets, eutectic Si is forced to grow around the Eu atoms. Therefore, there is a kinetic equilibrium of the adsorption of Eu atoms and the overgrowth by eutectic Si. In the case of high rates of Eu atoms adsorption, not only TPRE are poisoned but additional Eu atoms create further facets and growth directions *via* the IIT mechanism. IIT events ahead of the TRPE result in an entrainment of the segregated Eu atoms pushed ahead of the growing eutectic Si and subsequent to the formation of single Eu-rich clusters by segregated Eu atoms (Fig. 2c).

In summary, we have demonstrated, for the first time, excellent agreement between an atomic-scale experimental investigation on the distribution of Eu atoms and DFT calculations of the segregation behaviour of Eu atom within Si twins and its effect on the Si twinning. Both experimental and theoretical investigations reveal three different roles of Eu atoms during the growth of eutectic Si in Al-Si alloys. The proposed growth mechanisms can be also employed to interpret the growth of other modified Al-Si eutectic alloys.

## Methods

### Bulk sample preparation

Al-5 wt.% Si (hereafter marked as Al-5Si) alloy (wt.%, used throughout the paper unless otherwise noted) with 0.05 Eu addition was prepared using high purity (HP) Al (5N, 99.998), HP Si chips (6N), and Eu pieces (2N8, 99.8). 0.05 Eu addition was made using Al-5Si-2Eu master alloy produced from HP Al (5N), HP Si chips (6N) and Eu pieces (2N8). The chemical concentration of Eu was determined by inductively coupled plasma atomic emission spectrum (ICP-AES) apparatus. The P concentration (about 0.44 ppm) was determined with a Finnigan ELEMENT GD glow-discharge mass-spectrometer (GD-MS). The measured composition is listed in Table 2.

The Al-5Si-0.05Eu alloy was melted in an electric resistance furnace and the melting temperature was kept at about 720 °C. No degassing was performed prior to casting into a QuikCup sand mould. The cooling rate was evaluated to be 20 °C per min.

### TEM sample preparation

The samples for TEM investigation were prepared using the standard lift-out technique in an Auriga^®^ CrossBeam^®^ Workstation (Carl Zeiss SMT)^24^. The device combines a field emission cathode with an EDX system (EDAX, SDD Apollo 40) and a high resolution focused ion beam (FIB) (Orsay Physics Cobra Ga^+^ ion FIB) in one instrument. The FIB acts as a nano-scale scalpel to produce well-defined cuts within the material. Electron and FIB-columns are arranged at an angle of 54°, which permits a direct observation on the processing within the SEM. Slicing was performed to be perpendicular to the sample surface. The direction of cutting motion proceeds from the bottom to the top. After thinning the TEM samples, a low energy milling at a voltage of 2 kV was performed to minimize possible damages induced by Ga^+^ ions and redeposition of material on the surface of the lamella.

### HAADF STEM imaging and EELS spectrum

HAADF STEM imaging and EELS spectroscopy were performed using a Nion UltraSTEM100 aberration corrected dedicated STEM. The microscope was operated at an acceleration voltage of 100 kV and an electron probe convergence semi-angle of 31 mrad, which resulted in an estimated minimum electron probe size of 0.8 Å. The cold field emission gun of the microscope has a native energy spread of 0.35 eV. The HAADF detector semi-angles were 73–185 mrad and the spectrometer collection semi-angle was 36 mrad. High signal-to-noise HAADF images were acquired by means of integrating over stacks of 20–100 images, each with an exposure time of 0.5 s. Prior to integration, the image stacks were drift corrected using the SDSD plug-in^25^ for Gatan’s Digital Micrograph (DM) software.

All EELS spectra were de-noised using Principle Component Analysis (PCA) as implemented in the MSA plugin^26^ for DM. The de-noised dataset were thoroughly checked for any possible artefacts introduced by this process. EELS maps where then created by integrating the EELS signal of each edge over a suitable energy window after subtracting the preceding exponential background fitted with a power law. The Eu *M*_*4,5*_ edge (*M*_*4*_: 1131 eV, *M*_*5*_: 1161 eV) was integrated over a 100 eV range from the edge onset (1131 eV), where the background was subtracted fitting a 50–100 eV pre-edge region. Due to the presence of very low intensity Eu *M*_*3*_ (1481 eV), *M*_*2*_ (1614 eV) and *M1* (1800 eV) edges in the spectra, extra care was taken when mapping the Al *K* (1560 eV) and Si *K* (1839 eV) edges. For the Al *K* edge, the background was fitted in a range ≈1500–1550 eV and the edge signal was integrated over a region ≈1560–1600 eV, thus minimizing effects of the Eu *M*_*3*_ edge (1481 eV) on the background subtraction and the Eu *M*_*2*_ edge (1610 eV) on the integrated edge signal. For the Si *K* edge, the background was subtracted over a region ≈1720–1790 eV and the edge was integrated over a region 1839–1939 eV, thus minimizing any effect of the Eu *M*_*1*_ edge (1800 eV). (All EELS edges were identified following^27^)

The intensities of the EELS maps displayed in Fig. 2 were normalised for convenience in arbitrary units (on a scale of 0 to 1), and intended to show the relative spatial distribution of each element within the analysed area rather than a quantitative concentration. The maps are displayed on a false colour scale, so that within each map, a low intensity (black) corresponds to a lower relative concentration, while increased contrast (colour) corresponds to an increase in elemental concentration.

### Density functional theory calculations

The calculations were performed using VASP^28^,^29^,^30^,^31^ with projector augmented wave functions (PAW) and the exchange correlation functional PBEsol^32^,^33^. For Si, two *s* and the two *p* electrons were treated as valence electrons. For Eu, two s, six p and one d electron are treated as valence while 6 f electrons are added to the core. Results for an alternative configuration with two s and six p electrons in the valence and 7 f electrons in the core are provided in the supplementary information. The cut-off energies are taken from the default values for Si in the potential files supplied by VASP. Using these setting, the lattice parameters of Si were calculated to be 5.440 Å.

The Si TB structure was modeled as a Σ3<110> {111}, which was subsequently optimized by a γ-surface approach, where the two slabs constituting the TB were shifted with respect to each other. By this, the ground state of the TB is estimated. Details of the γ-surface approach and the supercell setup can be found in^34^ and the supplementary information.

## Additional Information

**How to cite this article**: Li, J. *et al.* The roles of Eu during the growth of eutectic Si in Al-Si alloys. *Sci. Rep.* **5**, 13802; doi: 10.1038/srep13802 (2015).

## Supplementary Material

Supplementary Information

## Figures and Tables

**Table 1:** Calculated segregation energy to the TB for four different cases.

	*E*_*seg*_ [eV per atom]	Bond lengths for Eu-Si [Å]
Case 1	−2.99	2.92–3.00
Case 2	−2.19	2.96
Case 3	−3.96	3.01–3.16
Case 4	−4.35	3.01–3.09

The bond lengths for Eu-Si extracted from the corresponding relaxed structures are also listed.

**Table 2:** The measured compositions of Al-5Si based alloy with 0.05 Eu addition.

Si	Eu	P (ppm)	Al
5.00	0.05	0.44	Balance

(wt.%).
