# Supplementary material for: The roles of Eu during the growth of eutectic Si in Al-Si alloys
Source: Sci Rep. 2015 Sep 2;5:13802. doi: 10.1038/srep13802 (PMC4557128; doi:10.1038/srep13802)
Supplement: Supplementary Information [file srep13802-s1.doc]

# The roles of Eu during the growth of eutectic Si in Al-Si alloys

Jiehua Li^1^*, Fredrik Hage^2^, Manfred Wiessner^3^, Lorenz Romaner^3^, Daniel Scheiber ^3^, Bernhard Sartory ^3^, Quentin Ramasse^2^, Peter Schumacher^1, 4^

^1^ Institute of Casting Research, Montanuniversität Leoben, A-8700, Leoben, Austria

^2^ SuperSTEM Laboratory, SciTech Daresbury Campus, Keckwick Lane, Daresbury, WA4 4AD, UK.

^3^ Materials Center Leoben Forschung GmbH, A-8700, Leoben, Austria

^4^ Austrian Foundry Research Institute, Leoben, A-8700, Austria

*Corresponding authors:

J.H. Li: Institute of Casting Research, Montanuniversität Leoben, A-8700, Leoben, Austria.

Tel.: +43-3842-402-3304; Fax: +43-3842-402-3302.

# Email address: [**jie-hua.li@hotmail.com**](mailto:jie-hua.li@hotmail.com)

**Supplementary Information**

**HAADF STEM imaging and EELS**

Figure S1a shows an example EEL spectrum averaged over the region indicated in the HAADF STEM image (Figure S1b). The spectrum was de-noised using principle component analysis and de-convoluted for thickness using the Fourier-Ratio method [1]. Clearly, the Al *K* edge is present in the spectrum, however, the intensity of this relative to that of the Si *K* edge is very small. As discussed in the main text, the detection of Al with EELS is likely due to the fact that the signal is averaged through the thickness of the sample, and some matrix may remain further in the depth of the lamella, giving rise to the observed signal.


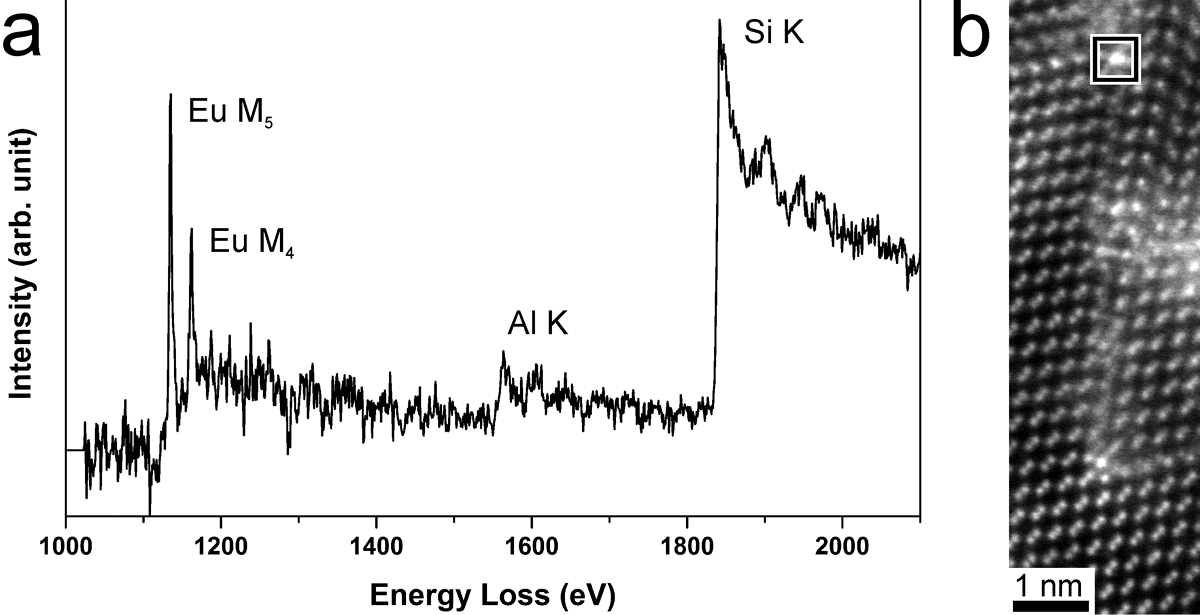


**Figure S1: (a) Example EEL spectrum averaged over the region indicated by the black square in the HAADF STEM image (b).**

**Density functional theory calculations**

***Methodology***

The Si TB structure was modeled as a Σ3<110>{111}. The supercell setup used is shown in

Figure S2.


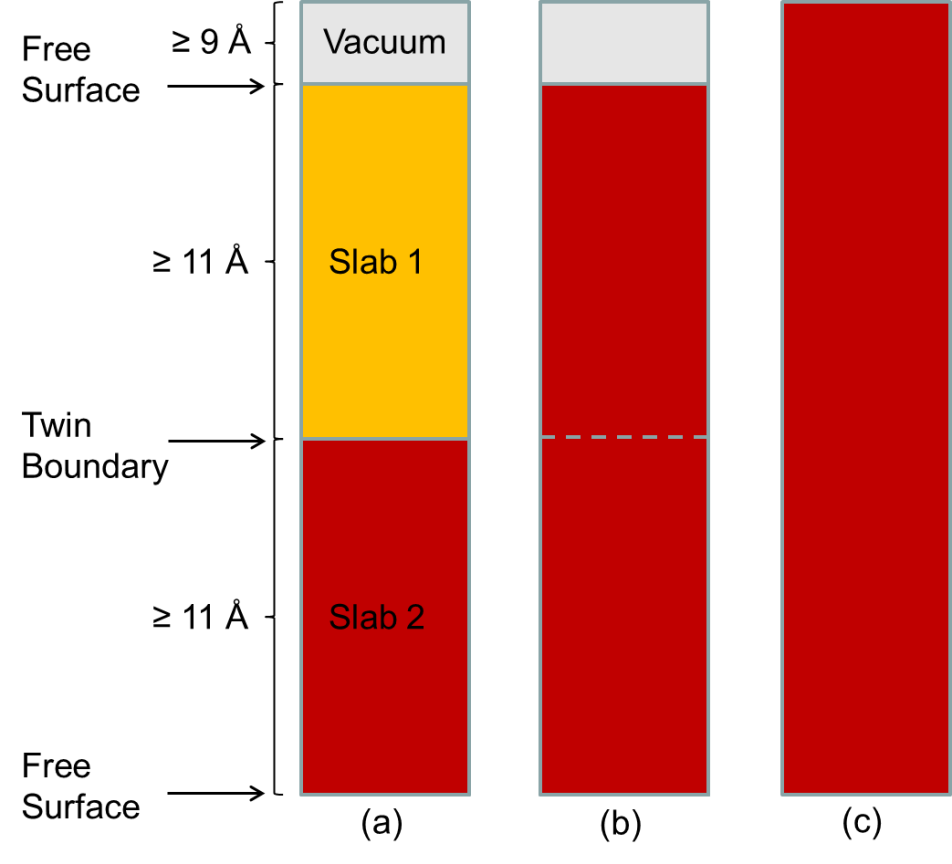


Figure S2: Supercell setup for TB calculations. (a) shows the TB cell containing a TB and two free surfaces, (b) shows the TB cell containing two free surfaces and bulk between the interfaces, (c) shows the reference bulk configuration. The length specifications are valid for γ-surface calculations.

Two slabs of 12 Å length are placed in a supercell and a vacuum layer of 9 Å length is inserted in the top part of the unit cell to decouple the bicrystal from its periodic replicas. The ground state geometry of the TB is obtained via a γ-surface approach [2]. The two slabs constituting the TB are initially shifted with respect to each other and a structural relaxation is carried out where all atoms are allowed to freely relax in the x, y and z direction. The unit cell is kept fixed, however, due to the vacuum inserted in the unit cell, the slab can relax normal to the TB. The result of this procedure is shown in Figures S2a and S2b. For every initial shift a geometry and corresponding energy is obtained. The structure of lowest energy is the ground state shown in Figure S2c. The geometry of the TB containing the Eu atoms (Cases 1-4 in Figure 3) was obtained with the same procedure.


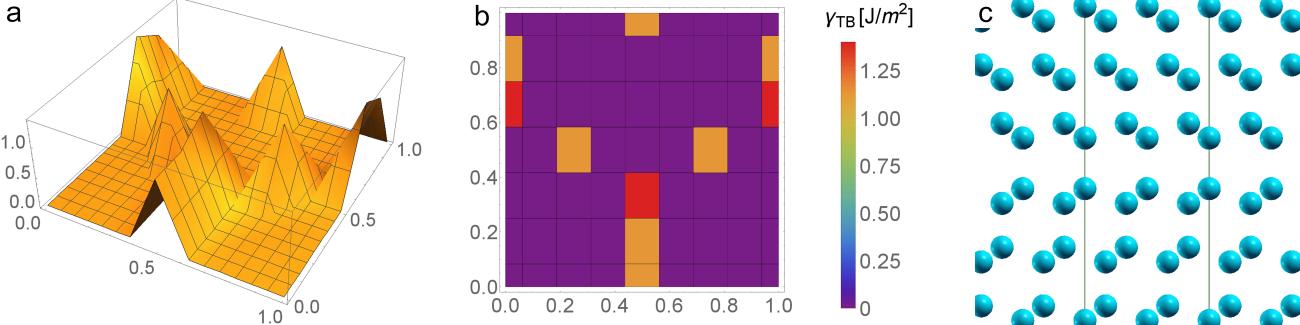


Figure S3: (a) γ-surface and (b) the plotted TB energy (γ_TB_) for pure Si TB, and (c) Ground-state structure for Σ3<110>{111} TB with zero shift.

To address the importance of expansion normal to the TB calculations were also carried out where the atomic layers at the free surface were not allowed to relax normal to the GB. In this case, the segregation energy was slightly higher, about 0.2 eVs for Case 3 and Case 4. Since the segregation energy amounts to 3 - 4 eVs, this effect is not dominant.

***Comparison with bulk setup and +2 oxidation state***

Analogous calculations in bulk Si were also performed and compared with the results in Si TB, as shown in Figure 3 and Table 1 in the main manuscript. It should be noted that Eu is in the +2 oxidation state. The calculations for the +2 oxidation state were carried out using the Eu_2 pseudopotential provided by the VASP code with 7 f-electrons in the core leaving 2 6s electrons, 1 5d electron and 6 5p electrons in the valence complex of Eu. The results are shown in Figure S4 and Table S1.

Similar to Figure 3, the structures presented in Figure S4 (top row) for bulk Si show a close resemblance with the structures in Figure 3 in the main manuscript. Also, the corresponding segregation energies[[1]](#footnote-2) ($E_{seg}^{Bulk}$) show the same relative energetic ordering and identify Case 3 and Case 4 as energetically most favorable structures, strongly indicating that it is the specific planar arrangement of Case 3 and Case 4 which leads to the large segregation energies of Eu atoms, rather than the twin geometry of bulk Si. Hence, if Eu atoms were arranged in a plane in the bulk, the same segregation energies would be obtained. Indeed, the reason why the planar arrangement occurs at the twin is not solid state segregation in the common picture (i.e. Eu atoms diffuse around in the solid and find energetically preferred positions at the interface). Instead, during solidification, Eu atoms are adsorbed ahead of solidification front, more precisely at the TPRE. It is the solidification process that creates the planar rearrangement.

After relaxation, the structures presented in Figure S4 (bottom row) obtained for bulk Si in a +2 oxidation state show similar structures when conparing with the ones obtained in the +3 oxidation state in Figure 3. For Case 2, it can be recognized that the Eu atoms are pushed further away, which is consistent with the higher lattice parameter that is obtained in the +2 oxidation state. The segregation energies are slightly lower than the one in Table 1 but show the same relative ordering. Again, Case 3 and Case 4 are energetically highly favored over Case 1 and Case 2.

Table S1: Calculated segregation energy to the bulk planar arrangement ($E_{seg}^{Bulk}$) and for the +2 oxidation state ($E_{seg}^{TB}$) for four different structural cases.

|  | $E_{seg}^{Bulk}$ [eV/atom] | $E_{seg}^{TB}$ [eV/atom] |
| --- | --- | --- |
| Case 1 | -2.18 | -3.15 |
| Case 2 | -1.91 | -2.46 |
| Case 3 | -3.81 | -4.37 |
| Case 4 | -4.33 | -4.57 |


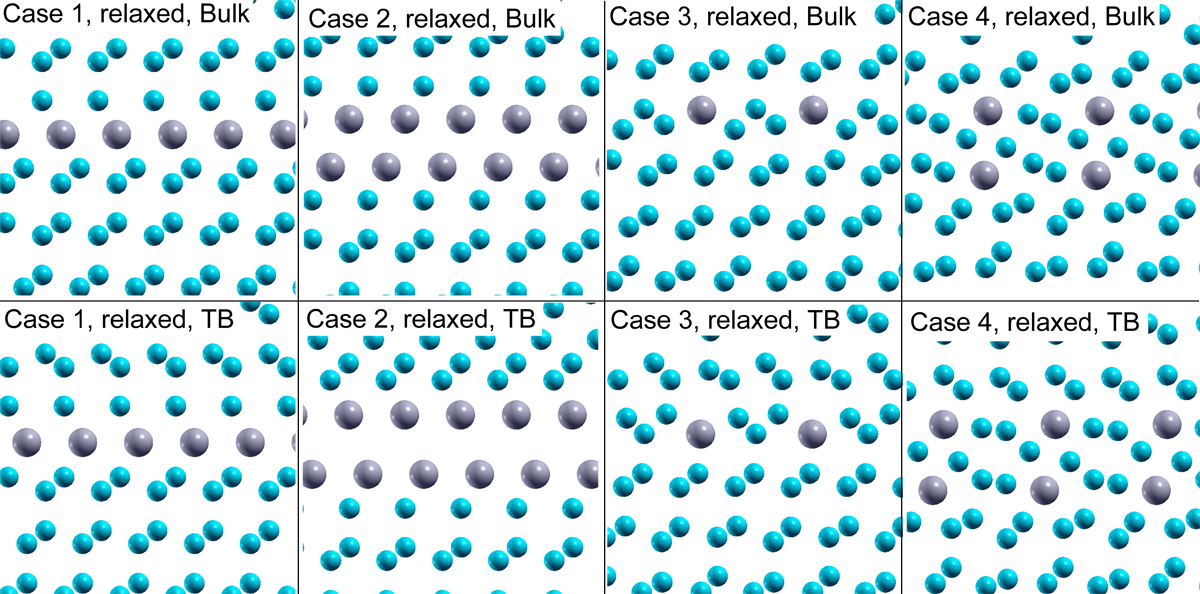


Figure S4: Structures for different cases with Eu in bulk planar alignment (top row) and with Eu at TB for an assumed +2 oxidation state Eu (bottom row).

***Strain energy analysis***

To understand the variations in segregation energy shown in Table 1 and Table S1, a strain energy analysis is carried out. Inserting a Eu atom in the Si lattice can be expected to strain the lattice due to the significant difference in atomic volume of Eu and Si. Figure S5 shows the Eu atom in a bulk Si environment before and after relaxation in the DFT calculation. The next-nearest neighbors Si atoms were observed to be pushed away from the Eu atom, increasing the bond length from 2.36 Å to 2.76 Å. The total energy of the unit cell decreases by about 3.72 eVs during relaxation.


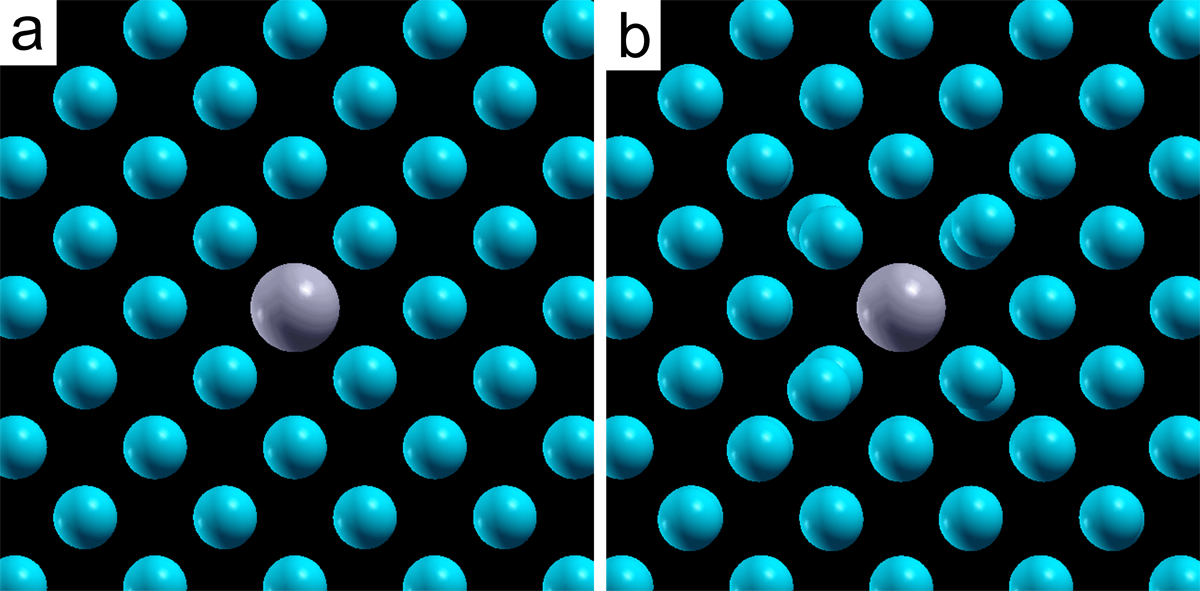


Figure S5: Representation of the Eu atom in the bulk Si structure before (a) and after (b) relaxing the atomic positions.

The strain energy stored in the lattice after relaxation is not readily accessible in DFT simulations. To extract this energy, in principle, a procedure has to be invoked where strain$\varepsilon_{kl}$ is calculated based on the observed distortion and the energy is calculated on the basis of a linear elasticity using equation S1

$E_{strain}=\frac{1}{2}C_{ij,kl}\varepsilon_{ij}\varepsilon_{kl}$ (S1)

where $C_{ij,kl}$are the elastic constants. Alterntively, the well-known (and widely used) sphere-in-a-hole model by Eshelby can be used [3]. The strain energy is obtained using equation S2:

$E_{strain}=\frac{2K^{Eu}G^{Si}(V^{Si}-V^{Eu})^{2}}{3K^{Eu}V^{Si}+4G^{Si}V^{Eu}}$ (S2)

where $K^{Eu}$ is the bulk modulus of Eu, $G^{Si}$ is the shear modulus of Si, $V^{Si}$ is the atomic volume of Si, and $V^{Eu}$is the atomic volume of Eu. For Si, $G^{Si}$= 80 GPa and $V^{Si}$= 20.0 Å^3^. For Eu in the +3 oxidation state, $K^{Eu}$=37 GPa and $V^{Eu}$= 33.3 Å^3^. For Eu in the +2 oxidation state, $K^{Eu}$=10 GPa and $V^{Eu}$= 46 Å^3^. With these values, $E_{strain}$= 0.5 eV for the +3 oxidation state and $E_{strain}$= 0.4 eV for the +2 oxidation state is obtained. The strain energy appears to be a moderate contribution compared to the differences in segregation energies, as shown in Table 1 and Table S1. This suggests that it is not the release of stored strain energy which leads to the large segregation energy and the large energetic differences between the different atom arrangements of Cases 1-4. This suggestion can be also supported by the following argument: when placing Eu atoms next to each other (as happens in the planar arrangements), linear elasticity predicts an increase in the strain energy per atom due to interactions between the compression centers. Hence, if the energetics were dominated by the strain energy, the segregation energy should be positive and always favor low Eu densities. This is not the case in our calculations. In contrast, a large energetic stabilization (up to 4 eVs) was obtained when arranging the Eu atoms in the plane, whether at the TB (Table 1) or in bulk Si (Table S1). These large segregation energies do not arise from strain effects but from chemical interactions. It is, therefore, the specific local coordination of Eu and Si atoms which leads to the high energetic stabilization of the Case 3 and Case 4 with respect to an isolated Eu atom dissolved in bulk Si.

***Reference***

1. Egerton, R.F. Electron Energy-Loss Spectroscopy in the Electron Microscope. Springer US, (2011).
2. Scheiber, D., Razumovskiy, V., Puschnig, P., Pippan, R. & Romanner, L. Ab initio description of segregation and cohesion of grain boundaries in W–25 at.% Re alloys. *Acta Mater*. **88**, 180-189 (2015).
3. Geng, W.T., Freeman, A.J. & Olson, G.B. Influence of alloying additions on grain boundary cohesion of transition metals: First-principles determination and its phenomenological extension. *Phys. Rev. B.* **63**, 165415 (2011).

1. Note that segregation energy here means the energy gain when taking isolated Eu atoms in bulk and arranging them in a plane. [↑](#footnote-ref-2)
